# The Hospital Nursing Supplement

**Published:** 1896-12-12

**Authors:** 


"The Hospital" Nursing Mirror.

Being the Extra Nursing Supplement of "The Hospital."

[Contributions for this Supplement should be addressed to the Editor, The Hospital, 28 & 29, Southampton Street, Strand, London, W.C., and should have the word "Nursing" plainly written in left-hand top corner of the envelope.]

### News from the Nursing World.

### PRINCESS LOUISE AT MANCHESTER.

The bazaar in aid of the funds of the Manchester Hospital for Consumption and Diseases of the Throat and Chest, was opened on November 25th by H.R.H. Princess Louise, who, with the Marquis of Lorne, had been staying at Knowsley with the Earl and Countess of Derby, and was a great success. Of course, the Princess's presence drew numbers of people to St. James's Hall, and the promoters of the bazaar must feel well content with the takings for the first day, which, including donations, amounted to something over £5,400. After the opening ceremony Princess Louise and the Marquis of Lorne were entertained at lunch at the Town Hall by the Lord Mayor of Manchester.

### QUEEN'S NURSES AT INVERNESS.

The report presented at the recent annual meeting of the Inverness Branch of the Queen Victoria's Jubilee Institute was an eminently satisfactory one, and some excellent speeches were made on the occasion. Dr. Norman Macleod, who moved the adoption of the report, bore eloquent testimony to the value and importance of district nursing amongst the poor, and impressed upon the "ladies, who seemed to divide their time very equally between cycling and afternoon teas," that they "did not exhaust their duty to the sick poor when they had contributed, say, half-a-crown to the funds of the Jubilee Institute." Nurse Nicholson, whose work in the district is universally appreciated, has been joined by another nurse during the past year, and thanks to the timely help of a friend, who has undertaken to pay the rent for two years, a suitable house has been taken and fitted up where the two nurses can live together, another friend arranging to pay for the wages of a servant for the same period.

### NURSING AT TOKYO.

The Society for the Propagation of Christian Knowledge in 1889 gave a grant to the Medical Mission at Tokyo, money which has now been exhausted, and Bishop Bickersteth has asked the Society for further help towards the objects of the mission. Two European nurses have been working there since last winter, and this necessary increase of staff has raised the annual expenses of the medical department to some £400, for which the mission is responsible.

### NORTH-EASTERN HOSPITAL FOR CHILDREN.

The North-Eastern Hospital for Children is appealing for funds. In a letter to the *Times* last week, signed by Lord Frederick Fitz-Roy (chairman), Sir Stuart Knill (vice-president), and the Bishop of Stepney, it is explained that the efforts made this summer in connection with the bazaar at Queen's Hall have resulted in freeing the hospital from debt, but that "unless £1,000 can be collected by the end of the year it must again become involved." Donations and new annual subscriptions are urgently needed. The hospital is situated in the midst of the vast and very poor population of Shoreditch, Bethnal Green, and Hackney, and ministers to the needs of some 16,000 children every year. Dwellers in the West End should find their way down the Hackney Road (an omnibus from the Bank goes past the door), visit this little hospital, and see for themselves the work it is doing. Mr. T. Glenton Kerr, the secretary (to whom subscriptions and donations should be sent at 27, Clement's Lane, E.C.), will, we feel sure, cordially welcome such visitors.

### THE LAY PRESS ON NURSES.

The writer of some startling statements on "Hospital Nurses at Golf" in last Saturday's *Daily Graphic* must be gifted with a remarkably brilliant imagination. It is, doubtless, quite correct that "the modern nurse seeks to chase cobwebs from brain and body" in outdoor exercise, but we should like to see the hospital where "strokes" and wonderful "puts" are discussed in the wards; "while more than one enthusiastic probationer has been reprimanded for 'teeing' a roll of bandage off the ward fender with a patient's crutch," or where practice for the links is carried on "with the handle of a student's walking-stick for 'lofting iron,'" through the medium of a "surgical dressing-basket," and "a ball compounded of lint scraps"! The modern nurse is by no means all she ought to be; but, whatever may come to pass in the future, at present it may be safely affirmed that such nonsense as this will make nurses worthy of the name indignant at the imputation on their common-sense and good feeling. The *Daily Graphic* article ends with a circumstantial account of a recent "foursome" at Wimbledon between representatives of the nursing staffs of two large London hospitals—West End *v.* East End. A great deal has been said and written on the abuse of nurses' uniforms of late; but if it be true that trained nurses—and one imagines, intelligent women—attached to "large London hospitals" can be so lost to the dignity of their profession as to masquerade on public links in a "compromise" of grey gowns, with grey capes lined with scarlet, and bonnets with long grey veils, the misuse would seem to begin very much at home. Golf, by all means, or any other healthy outdoor game for nurses, but let them leave their working garb behind them.

### FAIR WAGES.

The speech made by Lord Overtoun in presiding at the annual meeting of the Glasgow Sick Poor and Private Nursing Association seemed to show that that association is by no means ashamed, but rather boasts of the fact that the "profits of nursing in the West-end went to help the sick in the East-end," in other words, that the nurses on the private staff receive but a proportion of the fees they have fairly earned, while the rest of the money they have worked for is used to support the charitable side of the association, the district work amongst the poor. This is altogether wrong. The work of the district nurses should be well and fully supported on its own merits by the rich folk of Glasgow, while for the benefit of those who can pay for the best nursing the private staff should be entirely distinct, the nurses receiving their own fees less so much per cent. for working expenses. There are too many associations, excellent so far as the work done by the nurses is concerned, which thus make money out of their private staff for the benefit of a charity, which ought to be kept up by public support and not by the hard earned wages of nurses, of which they are by every law of justice entitled to the full advantage themselves.

### BIRMINGHAM AND MIDLAND HOSPITAL FOR WOMEN.

A BALL, under the presidency of Lady Newport, is to be held at the Grosvenor Rooms, Grand Hotel, Birmingham, on Friday, January 8th, 1897, in aid of the funds of the Birmingham and Midland Hospital for Women. Lady Newport has promised to be present, and the Lady Mayoress of Birmingham and Mrs. Chamberlain have kindly consented to join her in forming a reception committee. The hospital treats a great number of patients from Birmingham and the surrounding districts, and finds its subscription list by no means equal to the demands upon its funds.

### NEWS FROM IRELAND.

A CORRESPONDENT in Ireland, who tells us of Miss Shuter's appointment (noticed in another column) as Lady Superintendent of the City of Dublin Hospital, says that some regret is felt in Dublin that an Englishwoman should have been chosen for the post rather than a lady of Irish nationality. Miss Susan Beresford, who has just resigned after sixteen years' service, fulfilled the latter condition, being a member of the Waterford family, and a niece of the late Primate of All Ireland. Miss Shuter, we trust, will quickly overcome any feeling of disappointment of this kind, and Irish people are too instinctively courteous not to give the new matron a kindly welcome to her fresh work.

### MODERN NURSES.

"OLD-FASHIONED nurses," of whom, we may be thankful, there are still some amongst us, will be found to heartily endorse many of Miss Emma L. Watson's "Remarks on Modern Nurses" in this month's *National Review*. The numbers of utterly unsuitable women who are what might be called masquerading as nurses in the present day are a real grief and trial to their more sober-minded sisters. Miss Watson has only too much truth on her side when she says that, "owing to the thoughtless misbehaviour" of women who ought never to have been allowed to enter a hospital at all, there is a growing dislike to nurses among quiet people, who will put up with anything rather than introduce such an element into their houses. And it is no less true that this unfortunate state of things comes largely about because of those private nursing institutions which make large profits out of their nurses' earnings, and into which young and good-looking women, who have proved failures at their training-school, find easy access on account of an attractive appearance which will go down with the public. These things are very hard on the many honest, hard-working, and well-trained nurses who go quietly and soberly about their business, doing untold good, and whose value is certainly priceless. The moral of all this is that nurses who really love their profession, and are worthy of it, cannot be too careful to do and say nothing that shall add to the discredit which frivolous members bring upon it.

### A HOSPITAL MORTUARY.

FAR too little care is usually bestowed on the mortuaries of our hospitals, which are for the most part gloomy, miserable places, even when matters of cleanliness and sanitation are given proper attention. Signs of Christian reverence for the dead are altogether wanting in many cases. A hospital sister, speaking on the subject the other day, said: "It is terrible to take people to take their last look at some loved relation or friend amid these wretched, uncared-for surroundings," and her feeling is one shared by many. The mortuary chapel at the Hospital for Sick Children, Great Ormond Street, is a welcome exception to this state of things. It is a peaceful little place, large enough for several bodies to rest there at a time, if need be, and has been lately made quite beautiful with marble-lined walls whereon are a few simple, appropriate texts; and on the little altar at the east end is a cross and fresh flowers. It is lofty and well ventilated, and just what such a place should be. Evidently here fitting reverence and care is given to the dead.

### INCREASE OF STAFF FOR KENSINGTON INFIRMARY.

THE question of allowing the nurses at Kensington Infirmary additional leave has been under consideration for some time, and the sub-committee appointed to report on the matter have come to the conclusion that greater facilities for going out should be given, and would conduce to the better health of the nurses, a view shared by the Infirmary Committee, who recommended at a recent meeting of the Board of Guardians that application should be made to the Local Government Board for permission to make the change. The extra leave will necessitate an increase in the staff of four probationers. The report was adopted.

### A HAPPY THOUGHT.

MOTOR cars have been already pressed into the service of the hospitals. Lately, one of these novel carriages has been seen careering gaily about Ealing and the neighbourhood, offering passers-by "ten minutes' ride for a shilling," such shillings to be handed over to the local cottage hospital. A much-respected tradesman in Ealing was responsible for this happy thought, and it is said that quite a large sum resulted, for everyone was eager to try the motor car, and as the cottage hospital is deservedly a very popular charity, the shillings were paid in no grudging spirit. The hearty contempt of "them new-fangled things" manifested by the cabbies on the station stands was funny to see!

### SHORT ITEMS.

THE Queen has sent presents of old linen to the Royal Free, King's College, and London Hospitals, as well as to the East London Hospital for Children, Shadwell, and the Cancer Hospital, Brompton Road.—Princess Henry of Battenberg has added her name to the list of patrons of the Victoria Hospital for Children.—Miss Annesley Kenealy has severed her connection with the *Nursing Record*, of which paper she has been assistant editor for some time past.—A sale of bazaar goods was held in the out-patient hall of the North-Eastern Hospital for Children, Hackney Road, on Thursday, December 10th, being opened by Blanche, Countess of Rosslyn.—We are asked by Miss Isabel G. Lowndes (Secretary), to state that the office of the Northern Workhouse Nursing Association has been removed to 66, Barton Arcade, Manchester.

### Hygiene: For Nurses.

By John Glaister, M.D., F.F.P.S.G., D.P.H.Camb., Professor of Forensic Medicine and Public Health, St. Mungo's College, Glasgow, &c.

### XXXVI.—DISINFECTION AND DISINFECTANTS.

Disinfection includes those processes the object of which is, in one way or another, to destroy infective matter, and the nature of the process is largely regulated by the material which encloses, or bears, the infective matter. A disinfectant is an agent which destroys the power of infective matter to do further mischief. This term, unfortunately, has been used with but little discrimination to cover a number of agents of the most valuable and of the most valueless character. This has doubtless arisen from the nebulous conceptions which existed regarding the entity of infective matter. Often associated with disagreeable odours, it was supposed to be rendered inert by the use of deodorants. Since, however, the microbic constitution of infective matter became established, all such views have had to undergo revision, and the disinfecting power of any substance to be determined by its capability of destroying micro-organisms and their spores.

Disinfectants ought to be divided into three classes, viz., deodorants, antiseptics, and germicides. A deodorant (Lat.: *de*—from, *odor*—an odour) is something which destroys an odour, and is generally used to express that which deprives a bad odour of its disagreeableness. To this class belong camphor, musk, lavender, and like volatile odorous substances, tobacco, vinegar, eau de cologne, toilet vinegar, turpentine, terebene, permanganate of potash, or soda, as in Condy's Fluid, and others. In it may also be placed certain chemical substances which unite with their deodorant properties, that of acting germicidally or antiseptically, according to their strength. All deodorants act in one of three ways, viz. : (1) By submerging a bad odour by their superabundance, or by their more penetrating odour; (2) by oxidising odours, and converting them into new chemical substances; or (3) by changing a chemical substance having a bad odour into another substance which is odourless. Tobacco is a good illustration of the first; camphor, turpentine, and the essential oils of the second; and copper and iron sulphates (blue and green vitriol), of the third. The two former classes operate best in gaseous media, the last in liquids.

Antiseptics (Gr.: *anti*—against, *sepo*—to make putrid) are substances which, added to a putrescible substance, prevent the onset of putrefaction by lowering the virulency of the microbes contained in it. The length of time during which the condition of asepsis will continue depends upon the original strength of the antiseptic used. Usually the strength used is insufficient to do more than merely weaken the vitality of the organisms themselves, while it does not minimise the potency of their spores. If the strength, however, be sufficient, the substance acts as a germicide, whereas less strong as an antiseptic, and still less strong as a deodorant. To this class belong carbolic acid and its allies, since it is not sufficiently soluble in water to act as a germicide.

Germicides, or germ-killers, destroy the microbes of disease, some of them also destroying the spores. The potency of the germicidal agent depends entirely upon the strength in which it is used, and upon its applicability for this purpose, in the medium in which the microbes are located. To this class belong, of chemical substances, perchloride of mercury corrosive sublimate), biniodide of mercury, chlorine gas, chloride of lime (so-called), bromine vapour, and possibly chloride of zinc (as in Burnett's Fluid). In addition, and when used in sufficient volume for aerial disinfection, may be named sulphurous acid gas, iodine vapour, and in the disinfection of liquid or semi-solid substances, the sulphates of iron, copper, and zinc, cupralum, carbolic acid, sanitas, thymol, menthol, creosote, and the mineral acids, in sufficient strength.

Boric acid, boroglyceride, glacialine, sulphocarbolates of zinc and other metals, the sulphites and hyposulphites, are, at best, mild antiseptics.

Since our objective point in disinfection is the annihilation of microbes and their spores, it is essential that we should be acquainted with the substances which will, and those which will not, so act, since the market has been flooded with so-called disinfectants, which often by no means effect what is claimed for them. Indeed, seeing that effective disinfectants are of such public importance, they should be brought under the schedule of the Foods and Drugs Adulteration Acts, so that the public may be protected.

The following substances act efficiently as germicides when used of the named strengths:—

Table of Efficient Strengths of Disinfectants as Germicides.

| Disinfecting Substance. | Strength and Medium in which to be Used. |
|---|---|
| Mercuric chloride (corrosive sublimate) | 1 in 1,000 of water (70 grains per gallon). Solution to be aided by addition of common salt, and coloured with laundry blue. This may be used for disinfection of linen and liquid excreta. In 1 in 4,000 for washing woodwork and floors. |
| Mercuric iodide | 1 in 1,000 of water for infected linen and stools. 1 in 2,000 for hands and liquid excretions. 1 in 4,000 for woodwork and floors. Solutions ought to be rendered odorous by addition of small quantity of thymol, menthol, or eucalyptol. |
| Chloride of lime | 4 parts per 100 of water (6 oz. to 1 gallon of water) for solid and liquid excreta. When used for the former, to be permitted to soak for about one hour before vessel is emptied. 1 part per 100 of water is excellent for washing of hands after visitation to sick room and handling of patient. |
| Carbolic acid | 5 per cent. solution in water (1 in 20). Official strength used in Berlin, 1 in 18. To destroy bacteria, a 10 per cent. solution is needed. Water, however, will not dissolve so much. |
| Copper sulphate | 5 per cent. solution in water. To be used for disinfecting stools. Contact not less than one hour. |
| Zinc chloride (Burnett's fluid) | 1 in 10 of water (for stools), and as above. |
| Sulphurous acid gas (compressed in cylinders at three atmospheres) | Exposure for 12 hours to 4 volumes per cent. in a moist medium. 1½ lbs. of sulphur per 1,000 cubic feet of air-space=1·75 per cent. |
| Chlorine gas (in cylinders) | ·5 to 1 volume per 100 volumes of air. |
| Hydrochloric acid vapour | ·05 to ·1 volume per 100 volumes of air. |
| Bromine | 1 in 2,000 of water for solid excreta. Objectionable to user by reason of irritancy of vapour given off. |

### Christmas Gifts for the Hospitals.

We have to remind our readers that all contributions for The Hospital Christmas Clothing Fund should reach the office (28 and 29, Southampton Street, Strand) not later than the 16th inst., that is, next Wednesday. We have received one delightful bundle of warm stockings, and hope to see many a bulky parcel arrive in the course of the next week.

### Novelties for Nurses.

### NOVELTIES AT MESSRS. GARROULD'S.

Evidence is forthcoming abundantly on all sides of the rapid approach of Christmas, and never have the shops looked gayer than they are doing at present. Fascinating indeed is the display at Messrs. Garrould's, that well-known and deservedly popular emporium in the Edgware Road. Here nurses are extensively studied, and everything for their use is provided at a cost that makes one pause to inquire how it can possibly be done for the money. But it *is* done, and done well, too. The first thing that attracts attention on entering are the wallets and chatelaines, fitted with nickel plated instruments of the finest workmanship. The scissors and forceps can, if preferred, be those which unlock, a contrivance which enables them to be thoroughly cleansed and rendered aseptic. The Dora wallet, fitted complete for 22s. 6d., is calculated to rouse the most covetous thoughts in the beholder. There is also a comprehensive library of medical and other works useful to nurses, and many other things of like nature too numerous to mention. Passing to the drapery department, we confess to having quite lost our heart to the graceful and well-cut cloaks, of which the Angelus and Ellesmere certainly bear the palm. The latter in Melton cloth (25s. 6d.) is excellent value for the money, and is guaranteed to defy alike storm and sunshine. There are a variety of neat little bonnets, close-fitting in shape, trimmed with either velvet or ribbon, in connection with which we desire to draw attention to an excellent gossamer, called the "silver web," which is not only strong and durable, but preserves its appearance to the end. The caps are extremely dainty and pretty, the "Sister Dora," the "Pauline," and the "Sister Mabel" being among the number we most admired. Cuffs and collars are another indispensable item in a nurse's wardrobe, and Messrs. Garrould have of these a capital selection, to say nothing of the linen aprons, which are well cut and made from the best material. Boots and shoes form another useful branch of the establishment, and when we consider how important a matter it is to be comfortably shod, the advantage of being able to secure a thoroughly reliable article at moderate price cannot be over-estimated. Slippers for night duty (2s. 11d.), with twine soles, are a specialité which we have every confidence in recommending, as much for their durability, as their noiselessness and softness to the foot. It is almost superfluous to say anything about the enormous range of washing materials in every shade and quality for which Messrs. Garrould have acquired such a deservedly high reputation. Suffice it to state that development in this department is always going on, and that the linens and cottons of to-day are more durable, more beautiful, and more varied than they were ten or even five years ago, when striped galatea, grey, or navy-blue gingham were the one idea of the heads of our large nursing institutions.

### LINGERIE AT MESSRS. ROBINSON AND CLEAVER'S.

Messrs. Robinson and Cleaver are to be congratulated on the success that has attended their undertaking in Regent Street. It is but a year since they opened their branch establishment in London, and we have noted with pleasure the enormous development that has taken place in the business during that time. It is gratifying to find that the claims of nurses have been considered along with those of other customers, and that prices are quite within range of the slenderest purse. Aprons are one of their specialities, and a beautifully made article, hemstitched round the bottom, pockets and bib, is turned out at a price scarcely exceeding that which is paid for the same thing plainly stitched. The linen, too, is of first-rate quality, and likely to wear well. Cuffs and collars are made in all shapes and sizes of the finest linen, special designs being made to order. Of handkerchiefs there is a large assortment, some of the better qualities being wonderfully reasonable considering the beautiful finish and delicacy of the material. They are kept in two sizes, one for ladies and the other for gentlemen. Among the latter we observed two or three designs in the old-fashioned rimmed border, with narrow stitched hem, which so many prefer to the wide hemstitched article, but are so difficult to get nowadays. Those who are particular about this important adjunct to the toilette cannot do better than send to Messrs. Robinson and Cleaver for samples.

### Sketches of Hospital Life.

### THE DECKING OUT OF WILLIAM.

William Parkin was admitted one morning badly frost-bitten. It was during the great frost of 189—. On the previous evening he had presented himself at the workhouse clothed in a few rags. He was a huge man with a small peevish voice. The expression of his face was habitually aggrieved, and his smile was the smile of a martyr. By profession he was a "tinker." Before he was settled in bed he entered upon a harrowing description of the treatment he had received at the workhouse. The same evening he discovered that our butter had a flavour of candle grease. At breakfast, next morning, he was sarcastic about the tea. Luckily sister was a disciplinarian, or, as William sadly put it, "had a way with her." For the next two months he was a nuisance to everyone in the ward, including himself. He could not bear "hash"—a term he applied to everything but roast beef—said he had always been accustomed to lump sugar, and on one occasion demanded to see the doctor because his egg was not up to the regulation size. But all this is another story. When William was convalescent the question of providing him with attire had to be considered. It was a serious one, for he was six foot two inches in height and broad out of proportion. By much begging a miscellaneous outfit was accumulated—all but the boots. To buy these I went with a probationer to one of those shops which have most of their goods on the pavement. The shopman produced a cavernous pair, which he assured us were freaks as regards size. They were made up into two large parcels and proudly borne home. William saw them and shook his head. His fears proved well grounded—they were too small. Probably they are still in the matron's "museum," awaiting a tenant. A patient learned in clogs solved the difficulty, and a pair, rather suggestive of washtubs, were obtained, which William donned under protest. Once up, he had at last something he could reasonably grumble about. His coat in particular was risky, and he was not allowed to do anything in the wards which necessitated stooping. Once he upset the gravity of the senior physician by asking him in a grieved voice to look at his back.

At last William discharged himself, and about a week later a boy brought a large parcel and a very dirty note, "With Mr. William Parkin's compliments to Sister ——. He has no more use for the enclosed. The parcel contained his spurned attire, clogs and all. J. B. and M. D.

### Minor Appointments.

Royal Sea Bathing Infirmary, Margate.—Miss C. Jones has been appointed Staff Nurse at this institution. She received her training at Hitchin Infirmary.

Telfair Hospital, Savannah, U.S.A.—Miss Julia Tapping has been appointed Nurse to the children's ward at this hospital. Miss Tapping was trained at the Hospital for Sick Children, Great Ormond Street.

Castor Branch of Queen Victoria's Jubilee Institute for Nurses.—Miss Elizabeth Wilson has been appointed District Nurse for this branch. She was trained at the Royal Infirmary, Preston, remaining there for seven years, after which she took up private nursing in connection with the Royal Scottish Nursing Institution at Edinburgh. For three and a half years Miss Wilson was district nurse in Lancaster, subsequently taking charge of the infirmary, Beckham House, Gresham, Norfolk, for six months.

### Nurses in 1896—Their Quarters, Hours, and Food.

[These articles exhibit the actual condition of affairs in the spring of the present year.]

### POOR LAW INFIRMARIES.

### I.—TERMS OF TRAINING.

THE training now offered to probationers at the large Poor Law infirmaries, both in London and the provinces, where the superintendence of the nursing is vested in the hands of highly-trained and cultivated women, is valuable and excellent. Probationers are received somewhat younger at Poor Law infirmaries than at the voluntary hospitals. At Chelsea Infirmary, for example, candidates must be not less than 21 nor more than 30; at St. Marylebone the age considered desirable for probationers is from 22 to 32. Probationers are required to enter upon one or two months' trial, and usually sign an agreement to remain in the service of the Guardians for three years; at the two London infirmaries quoted above, of which the regulations are before us, the term of actual training being one year, service being continued for the remaining two as staff nurse. Certificates are given on completion of the full three years' engagement.

Regular instruction, lectures, and classes are given and held at these training schools by the medical superintendents and matrons, and examinations have also to be passed. The conditions of training at St. Marylebone are rather different from those at most infirmaries, the Guardians of that parish having undertaken to train probationers for the Committee of the Nightingale Fund, and, as at St. Thomas's Hospital, which trains Nightingale probationers, either retaining their services at the end of the first year or sending them to other hospitals where the matron herself is a Nightingale nurse, after the first year nurses may remain at St. Marylebone or, at the discretion of the Guardians, be sent to some other Poor Law infirmary approved by them to complete their three years' engagement.

Chelsea Infirmary is, we believe, the only Poor Law infirmary at which paying probationers are received. Here they may enter for six months' training, an arrangement which has been found to work exceedingly well, affording opportunities for many women who wish to make themselves acquainted with poor law work and can afford to pay for a short experience, and bringing into the infirmary a superior class of women, who help to raise the general tone and standard of the nursing.

### II.—HOURS OF WORK AND TIMES OFF DUTY.

In the Poor Law training schools the proportion of nurses to patients is smaller than in the voluntary general hospitals, but the absence of a large medical staff, coming at all hours, makes the work more regular, and the hours allowed off duty have, in many instances, been increased of late. It is still, however, by no means a universal custom for daily opportunities of going out to be given. At St. Marylebone, day nurses have several long days in the wards, being on duty from seven a.m. to half-past eight p.m., with half an hour each for dinner and tea, while on three afternoons a week only are probationers off duty from two p.m. to four p.m., with one evening from half-past five p.m. to half-past eight p.m.; nurses have also three afternoons from two p.m. to four p.m., and one evening from six p.m. to ten p.m., with on two Sundays out of three a half day, alternately from a quarter-past two p.m. to ten p.m., and a quarter to ten a.m. to a quarter-past one p.m. On these days, however, they are required to attend chapel when off duty. Head nurses are on duty from eight a.m. to half-past eight p.m., with leave on three afternoons from two p.m. to four p.m., one evening from six p.m. to eight p.m., and one from six p.m. to ten p.m. Sunday leave is the same as the staff nurses. All the staff have one day off in the month. Night nurses' hours on duty are half-past eight p.m. to half-past eight a.m. Probationers and staff nurses have three weeks' holiday in the year, head nurses four weeks. Night nurses have an hour and a half's leave every day, and one night a month.

At Chelsea Infirmary the daily hours are less, probationers and staff nurses being on duty from seven a.m. to half-past seven p.m., charge nurses from eight a.m. to half-past seven p.m. Three hours are allowed off duty four times a week and in summer it is possible to go out every evening. Night nurses are on duty from half-past seven p.m. to half-past seven a.m., with daily leave of absence. Monthly days off are given.

At Lewisham Infirmary daily leave of at least one and a-half hours is allowed, head and staff nurses having a weekly half day and a monthly whole day off duty; probationers a half day and a whole day each month.

### III.—MEALS.

Regular diet tables are drawn up for all "officers" under the Poor Law, everything in the way of rations being allowanced per head per week. That for St. Marylebone may be taken as a sample, the following being the weekly allowances for the nursing staff: 7 lb. bread, 7 lb. meat (uncooked and including bone), 7 lb. potatoes, 1 lb. flour, 1 lb. rice (or ½ lb. currants, sago, or tapioca), ¾ lb. butter, ¾ lb. bacon, ½ lb. cheese, 1 lb. loaf sugar, ½ lb. moist sugar, 7 pints milk, 7 eggs, 4 oz. tea, 8 oz. coffee, 3 oz. jam, 3 oz. syrup, fish once a week, vegetables daily, 7 pints ale (Kops' Ale, aerated waters, or milk, as preferred); pepper, mustard, vinegar, pickles, and sauces, salt and fruit (for cooking) as required.

All meals, except night nurses' ward meals, are laid in the dining-rooms, half an hour being the usual time allowed, sometimes three-quarters for dinner.

### IV.—SALARIES AND UNIFORM.

Pay for nurses under the Poor Law averages much the same as at voluntary hospitals for probationers; staff and charge nurses are, perhaps, not so well off, though the amount varies in the different Unions. Probationers are not paid during their first year at the Chelsea Infirmary, receiving only £3 10s. as "beer money." The second year they are paid £18, and the third £20, in each case with the addition of the £3 10s. Salaries for staff and charge nurses rise from £20 to £40 for night sisters and the home superintendent.

A liberal amount of uniform is supplied at most infirmaries, including sometimes besides dresses, caps, and aprons, cuffs and collars also; and all necessary washing is found. These large institutions have well-appointed laundries wherein all the washing for the staff as well as for the patients is done. A special item in the uniform supplied is usually a large, warm shawl, a necessary comfort in crossing the airy bridges which connect the infirmary blocks.

### V.—NURSES' QUARTERS.

The accommodation provided for the nursing staffs at a modern Poor Law infirmary is usually excellent. Good-sized single bed-rooms are the rule in all the more recently-erected homes, and there are no prettier or more thoroughly cosy sitting-rooms and recreation-rooms for nurses in London than those at St. Marylebone, St. Pancras, and Chelsea Infirmaries. At the last-mentioned infirmary the new home opened a few weeks back is quite a model of comfort and almost luxury, with its amply-furnished rooms and electric light. Ward sisters and head nurses have bed-sitting-rooms with fire-places, and usually a separate dining-room. At those infirmaries we have visited the meals are nicely served, and the comfort of the nurses at meal times and in off-duty hours well cared for. The standard of comfort and convenience in these homes is high, and it is specially good that the system of giving each nurse and probationer an entirely separate room has been accepted as necessary and desirable.

### Notes from South Africa.

From a Correspondent.

A very large proportion of the population of the Cape Colony is, of course, Dutch. There are many good old Dutch families whose names are known and respected all over the Colony, and who perpetuate the best traditions of their forefathers. On the other hand, there is a large class of low Dutch, who seem to deteriorate rather than to improve as years go on. They are indolent, unenterprising, non-progressive, and amazingly ignorant; and they intermarry largely with the coloured people. In sickness these people are quite as helpless, as superstitious, and as comfortless as the natives. They often live in hovels no better than native huts, and have not the most elementary ideas of nursing or doctoring. They will leave a patient lying in the same sheets, and in the same night-dress, day after day, and never think of washing him; and they invariably keep every door and window in the sick-room tightly shut, day and night, and stop up every chink and cranny with dirty old rags.

Many years ago, in a terrible epidemic of small-pox, an English farmer was acting as doctor and nurse in a large district where, at that time, there was neither the one nor the other. He went to one of these miserable Dutch farms to see a girl who was very ill with small-pox. When he entered the room where she lay, he was almost knocked down by the foul odour of it. Every chink and corner was tightly stopped with rags, door and window were kept shut, the room, the bed, and the unhappy patient were filthy. He went straight to the window and threw it open, setting the door wide open at the same time, so that a fresh draught of air blew through the room. The poor girl was dying, and he could do nothing for her; but he said he should never forget the look of relief and gratitude she turned on him when he admitted the fresh air. He discovered afterwards that the moment his back was turned the people of the house went in and closed the window and the door, and stuffed them up with rags again! Fresh air and water were like poison to them.

One of these ignorant Dutchwomen came to help an English lady, whose baby was suffering from terrible convulsions. The Dutchwoman was very big and stout, as many of them are, and filled up quite a space in the little bed-room, but every time the child had a fit she was so frightened that she ran out of the room. The father of the child, seeing this, observed to his wife, "Well, this old ironclad won't be of much use to you."

When, later on, the poor little baby was dying, with fearful struggles, another Dutchwoman, who was also there, said to the mother, "Get a soft pillow quickly; the spirit cannot escape while it lies on a hard pillow." The pillow was of hen's feathers, and this woman firmly believed that the child could not die until it was replaced by a pillow of goose feathers!

If anyone wishes to see a practical example of the evils arising from intermarriage of cousins, he can find it among the Dutch people all over the Cape Colony. This fatal error is not confined to the lower classes of Dutch. It arises from the great desire in all the Dutch families to keep their property in the family, and to effect this object, cousins marry each other for generation after generation. What is the result?

In one lunatic asylum there are, or have been, as many as six or eight patients of the same name, all belonging to branches of the same family, and generally having relations in other asylums. The family themselves own that it is the result of intermarriage.

In a certain valley there were several farms all belonging to different members of one Dutch family. The children of these farmers intermarried, and the grand-children, and the great-grandchildren, until the valley was full of idiots and lunatics, and the Dutch minister absolutely refused to marry any more of these cousins. "Go out," he said, "into other parts of the country, and get yourselves husbands and wives from other families."

Some time ago, the Dutch members of the Cape Parliament, seeing how the Dutch people were deteriorating from this custom of intermarriage, brought a Bill before Parliament to render the marriage of cousins illegal. They used their utmost efforts to force it through, but without success.

It is to be hoped that one day such an Act will be passed, not only in the Cape Colony, but in every civilised country; for there can, I think, be no reasonable doubt that a great deal of insanity and idiocy, to say nothing of other diseases, might in this manner be prevented.

### Where to Go.

St. Martin's Town Hall.—Mr. Bancroft has promised to give a reading of Dickens' "Christmas Carol" in aid of the funds of Charing Cross Hospital on Friday evening, January 22nd.

The Hospitals Association.—A general meeting will be held at the Society of Arts, John Street, Adelphi, W.C., on Monday, December 14th, at eight p.m. The chair will be taken by Mr. Boulnois, M.P., and a paper on "The Poor Law Officers' Superannuation Bill" will be read by Mr. H. Elwin Harris, medical superintendent of St. Saviour's Infirmary, S.E.

Royal British Nurses' Association.—The secretary requests us to state that an attractive programme is being arranged for the annual conversazione on Friday, December 18th. Mademoiselle Guilia Ravogli, Herr Johannes Wolff, Miss Agnes Valleris, Miss Honor Brooke, Miss Helen Jackson, and other eminent professional artistes have most generously given their services for the occasion. Members desirous of receiving their badges from H.R.H. the President are requested to communicate with the Secretary without delay.

### Appointments.

Evesham Cottage Hospital.—Sister Mary Hipkins has been appointed Matron at this hospital. She was trained at the Queen's Hospital, Birmingham, where she remained for five years, latterly as sister-in-charge of the accident ward.

Monkwearmouth and Southwick Hospital.—Miss E. F. Deakin has been just elected Matron of this hospital. Miss Deakin was trained at the Newcastle-on-Tyne Royal Infirmary, where she was subsequently charge nurse and night superintendent, and she has also held the post of Matron at the Dene Home Hospital, Newcastle-on-Tyne.

City of Dublin Hospital, Dublin.—Miss Helen Shuter was last week appointed to fill the post of Lady Superintendent at this hospital. She was trained at St. Bartholomew's Hospital and St. Thomas's Hospital, subsequently holding the position of sister at the latter institution, and a similar appointment at Paddington Infirmary.

Camelon Public Health Hospital.—Miss Helen R. Maxwell, who was trained at the Glasgow Royal Infirmary, has been appointed Nurse Matron at this hospital. Miss Maxwell was head nurse at Dr. Halliday Croom's Home, in Edinburgh, after which she joined the Scottish Branch of the Queen's Jubilee Institute, and worked as Queen's Nurse at Larbert.

Margam Urban District Council Isolation Hospitals.—Miss Grace Annie Bennett has been appointed Matron on November 24th. We have received no particulars of her training. Previous appointments held by Miss Bennett have been the matronship of the Home Hospital, Buckhurst Hill, and the post of charge nurse at Eston Hospital, Middlesbourgh.

Telfair Hospital, Savannah, U.S.A.—Miss Katharine Short has been appointed Matron of this hospital. She received one year's training at the Hospital for Sick Children, Great Ormond Street, and six months at the Samaritan Hospital for Women, Nottingham, then going for three years to Westminster Hospital. Subsequently Miss Short held successively the posts of night sister, out-patient sister, and ward sister at the Children's Hospital, Great Ormond Street. Miss Short has just completed her training in midwifery, and obtained the L.O.S. diploma. She possesses excellent testimonials from each of the hospitals where she has worked.

### Pirated Advertisements.

We have received the following letter and would refer our readers to "The Hospital" Nursing Supplement for January 26th, 1895, page cxxxiv; to the Nursing Supplement for February 23rd, 1895, "Everybody's Opinion," and to the Nursing Supplement for February 29th, 1896, page cxc, where they will find evidence that the practices complained of have prevailed for a considerable period. Further comment on our part should be unnecessary, as every intelligent nurse will now be in a position to protect herself from annoyance or loss by never answering pirated advertisements:—

"December 6th, 1896.

"Dear Sir,—Is there no way in which busy superintendents may protect themselves from this sort of thing? A few weeks ago I advertised a post as vacant in The Hospital, and during the next week received ninety-four replies, and out of that number selected the candidate to fill the vacancy, when to my dismay I see my advertisement copied by the *Nursing Record* in the next week's issue, so that it would reach any readers that paper may have after the post was filled up. I at once wrote to the Editor of the *N.R.* (but so far have received no apology), pointing out the cost, vexation, and trouble to the nurse of applying for a post already given away, and to the already overburdened superintendents of large and busy hospitals in receiving such applications, replying to them, and returning their testimonials. This is the second time that I personally have suffered from a pirated advertisement, and should you be able to ventilate the question of how a nuisance of this kind may be stopped it would be a boon to many busy people.—Truly yours,

"Lady Superintendent of a Hospital of 166 Beds."

### Everybody's Opinion.

### THE NURSES AND QUEEN VICTORIA.

Sister Ruth writes: I was very pleased indeed to see in The Hospital for November 28th a letter from a Pension Fund nurse saying that she thinks it a pity that the maximum amount fixed for nurses to pay annually to the Junius Morgan Benevolent Fund should be one shilling only. I certainly think it should be two, except, perhaps, for the younger nurses, who naturally cannot afford to pay more than one, and in many cases not that even. Trusting that all those nurses who can afford to do so will at least try.

### THE PREVENTION OF INSANITY.

A Correspondent writes; There is a great deal of truth in the letter published under this heading in The Hospital for November 28th. I doubt, however, if the general public will be very keen in the matter of instruction with regard to the prevention of insanity. The late Dr. Hack Tuke published in 1878 (Macmillan) a very readable book, "Insanity and its Prevention," on this subject, intended for lay readers as well as medical men; but, as I do not think it got beyond a first edition, it would seem not to have been a conspicuous success. Still, in the eighteen years that have elapsed a new generation has been growing up, and more interest may now be felt in questions of this character than formerly.

### RURAL NURSING.

"A Member of the Metropolitan Nursing Association in Bloomsbury Square from the Beginning" writes: I have read with some surprise the resolution passed at the recent meeting held at Stafford House, viz., "the establishment of a centre in London where information on rural nursing throughout Great Britain may be obtained." We have always supposed that such a centre exists at St. Katharine's Royal Hospital, Regent's Park, and as the Queen's Jubilee Institute is about to be developed, as we hope, to cover all England, I am unable to see the advantage of starting another centre, which can only lead to confusion. Surely the multiplication and over-lapping of similar institutions is one of the great evils of our present system of philanthropic work.

*** Our correspondent has evidently misunderstood the resolution she quotes. The object is to establish a centre to enable the authorities of County Nursing Associations to inter-communicate and so to provide that these organisations which are for the provision of so-called "County Nurses," are thoroughly well worked on something like an identical system, and are so placed in a position to enable the Council of the Queen's Jubilee Nurses to undertake the inspection of their nurses all over the country.—Ed. *T. H.*

### A HOSPITAL WITHOUT MUSIC.

"A Hospital Visitor" writes: It is the happy custom in the present day—and one which prevails more largely every year—to make the interiors of our hospitals as bright and cheerful as circumstances will permit. To this end pianos and American organs, musical boxes, or harmoniums are to be found in almost every hospital ward, and visitors with musical gifts are welcome, indeed, to give an hour's amusement and pleasure to the patients. The hymns at prayers and on Sundays, too, are a great source of pleasure, and a hospital sister regards her ward piano or other instrument as a never-failing resource by which many a weary hour may be beguiled. But I find there is one hospital in London into which the sound of music never enters, where it is altogether "taboo." And why? Because the patients are too seriously ill? No, for it is the Royal Eye Hospital, Southwark, where for the most part the patients are not at all ill, and being unable to amuse themselves with reading, &c., are more than usually dependent on outside help to cheer their days in hospital. The reason, I understand, is because one of the medical staff and member of the committee, an eminent oculist (Professor McHardy) has caused to be added to the rules of the hospital one which prohibits within its walls music of all kinds, and even the nurses are not permitted to have a piano in their own sitting-room. I hear, too, that a kind friend of the hospital has offered this year to provide a Christmas tree and gifts for the children and other patients, but this also has been forbidden. Evidently the efforts which, at most hospitals, make Christmas a time to be remembered with pleasure and gratitude by those under treatment in its wards, are at a discount at the Royal Eye Hospital.

### POOR LAW OFFICERS' SUPERANNUATION BILL.

Mr. J. S. Toogood, M.D., Medical Superintendent of the Lewisham Infirmary and hon. secretary of the Medical Superintendents' Society, writes: I think it may interest your readers to know that the Medical Superintendents foresaw the injury likely to be inflicted upon infirmary nursing by the "Poor Law Officers' Superannuation Acts." At a special meeting held on March 16th, 1895, at the Medical Society's Rooms, it was resolved to acquaint Mr. Walter H. Long, M.P., who then had charge of the Bill, "that in the opinion of the Medical Superintendents of the infirmaries, fever hospitals, and asylums, all contributions to the superannuation fund by subordinate officials be refunded without interest on their voluntary resignation." Mr. Long replied that he was afraid "the change would not be generally acceptable." Immediately after a meeting held on February 15th, 1896, the same resolution was sent to Mr. J. Bailey, who had charge of the present Bill. Mr. Bailey did not even acknowledge the receipt of the letter, although I know he received it. Section 8 provides that "an officer or servant . . . who loses his office by reason . . . of any cause whatever other than his own misconduct or voluntary resignation shall be entitled to receive . . . a sum equal to the amount of all his contributions." It appears to me that probationers, who are all appointed for a certain period, will, at the expiration of that time be entitled to the return of their contributions provided they do not remain in the service of any board of guardians. Scrubbers and washers, many of whom are over 65 years when they commence their service under boards of guardians, could, I feel sure, be put outside the pale of the Act by appointing them for limited periods, and reappointing them, say, at intervals of three months.

### For Reading to the Sick.

### THE SEASON OF ADVENT.

**Motto.**

"God with us."

**Verses.**

Hark! what a sound, and too divine for hearing,
Stirs on the earth and trembles in the air!
Is it the thunder of the Lord's appearing?
Is it the music of His people's prayer?
Surely He cometh, and a thousand voices
Shout to the saints and to the deaf are dumb!
Surely He cometh, and the earth rejoices,
Glad in His coming, Who hath sworn, "I come!"

—*F. Myers.*

O quickly come, dread Judge of all,
For, awful though Thine advent be,
All shadows from the truth will fall,
And falsehood die, in sight of Thee.
Oh! quickly come; for doubt and fear
Like clouds dissolve when Thou art near.

—*Tuttiett.*

Hark, a thrilling voice is sounding,
Christ is nigh it seems to say;
Cast away the works of darkness,
O ye children of the day.

Wakened by the solemn warning
Let the earth-bound soul arise;
Christ, her Sun, all ill dispelling,
Shines upon the morning skies.

—*E. Caswall.*

**Reading.**

"Be ye also ready; for in such an hour as ye think not the Son of Man cometh."

"I came down from Heaven, not to do Mine own will, but the will of Him that sent Me."—*John* vi. 38.

"I am come that they might have life, and that they might have it more abundantly."—*John* x. 10.

"Almighty God, give us grace that we may cast away the works of darkness, and put upon us the armour of light, now in the time of this mortal life in which Thy son, Jesus Christ, came to visit us in great humility; that in the last day, when He shall come again in His glorious Majesty, to judge both the quick and dead, we may rise to the life immortal, through Him, who liveth and reigneth with Thee and the Holy Ghost, now and ever."

Now of the result of that great overthrow, of the new heaven and new earth which are to come forth at God's command from the ruins of the old, what have we to say? But little, for God has told us little. "It doth not yet appear what we shall be." It could not yet appear. The mortal cannot comprehend immortality, nor the corruptible conceive of incorruption. The language which foretells these changes becomes accordingly mystic and symbolical. The vision of a city whose gates are precious stones, and whose streets are gold, that needs not the light of the sun, nor of the moon, through whose streets flows a mystic river, by whose banks grows a mystic tree of life—what does it tell us, save that the language which men speak on earth has no words in which it were possible to reveal the joys of Heaven? . . . One thought, however, respecting our future life, we can with some distinctness grasp; namely, that it must be a state of infinite progress; a life, not as we too often think of it, of progress arrested, a life in which humanity once, and once for all perfected, has before it only an eternity of virtuous repose; but rather one of intense and incessant activity. The promise of eternal life necessarily implies this, for life is something more than mere existence. Life, in its truest meaning, is the highest and happiest manner of being; it is existence with every power of our nature in its fullest, freest exercise.—*Archbishop W. C. Magee.*

### Notes and Queries.

The contents of the Editor's Letter-box have now reached such unwieldy proportions that it has become necessary to establish a hard and fast rule regarding Answers to Correspondents. In future, all questions requiring replies will continue to be answered in this column without any fee. If an answer is required by letter, a fee of half-a-crown must be enclosed with the note containing the enquiry. We are always pleased to help our numerous correspondents to the fullest extent, and we can trust them to sympathise in the overwhelming amount of writing which makes the new rules a necessity. Every communication must be accompanied by the writer's name and address, otherwise it will receive no attention.

*St. Luke's Hospital, New York.*

(59) Information wanted about this hospital.—*Nurse Talbot.*

Write direct to the secretary asking for the particulars you require.

*Nursing in Johannesburg and Malta.*

(60) Can you tell me if there would be a good opening for a private nurse in either of the above-mentioned places, and if there is a hospital at Malta where English nurses are employed?—*N. M. K.*

You should obtain information from someone living at these places. Doubtless the English matron at the Johannesburg Hospital might be able to give you such, but in our opinion nurses will be very foolish to attempt private nursing on their own account in South Africa or any other foreign land, unless they have independent means or sure and certain prospect of work. There are English nurses, we believe, at the Station Hospital, Valletta, Malta.

*Leper Island.*

(61) Can you tell me how to communicate with the authorities of the Leper Island?—*F. G.*

Of what island are you speaking? If you mean Robben Island, Cape Colony, you will find the names of the authorities on p. 714 of Burdett's "Hospitals and Charities" for 1896, and as for means of communication, why not write to the matron of the leper wards for any information you may desire? If correspondents require replies to queries by post, an order for 2s. 6d. must be enclosed. See note at head of this column.

*Training.*

(62) Please tell me how a lady can get into a London general hospital to train as a probationer?—*Coddenham.*

Apply to the Matrons of the various London Hospitals, of which you will find a complete list in Burdett's "Hospitals and Charities" (Scientific Press, 28 & 29, Southampton Street, Strand, London, W.C.), for particulars of the training, and a form of application for the candidate to fill in, should she be eligible under the rules of the institution. During the last six months full particulars of the terms of training at the principal London Hospitals have been given in The Hospital in the articles headed "Nurses in 1896."

*Registered Nurses' Association.*

(63) Is there an institution of private nurses known as the "Registered Nurses' Association," where I hear that nurses take their own fees?—*Nurse.*

There is a "Registered Nurses' Society," 269, Regent Street, W., and a "Society of Chartered Nurses," 24, Princes Street, Cavendish Street Square, W., the members of these societies being on the list published by the Royal British Nurses' Association. The address of the latter Association is 17, Old Cavendish Street, W.

*Nursing in South Africa.*

(64) I should like to get an appointment in a South African hospital. How should I set about it?—*Nurse D.*

Many nurses wish the same thing, but there is no such demand for English nurses in South Africa as our readers appear to imagine. You can only watch for an advertisement in our columns or write to the matrons of the various hospitals in South Africa, of which you will find a list in Burdett's "Hospitals and Charities."

*Mental Nurses.*

(65) I am a mental nurse, and wish to be registered. To whom should I apply, and what are the fees?—*Abbott.*

You seem to be labouring under some misapprehension. There is no general registration for any nurses, mental or otherwise. Various societies and associations keep "registers" of their own, and the Medico-Psychological Association gives certificates to asylum nurses who successfully pass its examination, the best qualification which such nurses can obtain at present. You should write to the secretary of the association, 11, Chandos Street, W.

*Electrolysis.*

(66) Can you give me some information regarding electrolysis as a means of removing superfluous hairs from the face?—*A.H.O.G.*

You should consult a medical man.

*Navy Nursing Service.*

(67) Will you tell me how admittance can be obtained to Netley Hospital as a probationer? I should also like to know what are the qualifications and conditions of training at London hospitals where no premium is required.—*Netley.*

You had better get and read "How to Become a Nurse" (from the Scientific Press, 28 & 29, Southampton Street, Strand, London, W.C.), in which book you will find replies to all your questions. Candidates for the Naval Nursing Service, as for the Army Nursing Service, are required to produce certificates of training at a good general hospital.

*Training.*

(68) Can you tell me of any hospital where probationers are taken at the age of 19?—*Probationer.*

A few children's hospitals take probationers as young as 19. You will find a list with all such particulars given in "How to Become a Nurse" (Scientific Press, 28 & 29, Southampton Street, Strand, London, W.C.).

